# The Oxytocin Receptor Gene (*OXTR*) in Relation to State Levels of Loneliness in Adolescence: Evidence for Micro-Level Gene-Environment Interactions

**DOI:** 10.1371/journal.pone.0077689

**Published:** 2013-11-04

**Authors:** Eeske van Roekel, Maaike Verhagen, Ron H. J. Scholte, Marloes Kleinjan, Luc Goossens, Rutger C. M. E. Engels

**Affiliations:** 1 Behavioural Science Institute, Radboud University Nijmegen, Nijmegen, The Netherlands; 2 School Psychology and Child and Adolescent Development, KU Leuven - University of Leuven, Leuven, Belgium; George Mason University/Krasnow Institute for Advanced Study, United States of America

## Abstract

Previous research has shown that the rs53576 variant of the oxytocin receptor gene (*OXTR)* is associated with trait levels of loneliness, but results are inconsistent. The aim of the present study is to examine micro-level effects of the *OXTR* rs53576 variant on state levels of loneliness in early adolescents. In addition, gene-environment interactions are examined between this *OXTR* variant and positive and negative perceptions of company. Data were collected in 278 adolescents (58% girls), by means of the Experience Sampling Method (ESM). Sampling periods consisted of six days with nine assessments per day. A relation was found between the *OXTR* rs53576 variant and state loneliness, in girls only. Girls carrying an A allele had higher levels of state loneliness than girls carrying the GG genotype. In addition, adolescents with an A allele were more affected by negative perceptions of company than GG carriers, on weekend days only. No significant gene-environment interactions were found with positive company. Adolescents carrying an A allele were more susceptible to negative environments during weekend days than GG carriers. Our findings emphasize the importance of operationalizing the phenotype and the environment accurately.

## Introduction

The need to belong hypothesis states that every human being has an innate drive to form and maintain a certain number of relationships with other humans [1]. When this need is not fulfilled, people can experience social pain, for example in the form of feelings of loneliness. From an evolutionary perspective, feelings of loneliness can be functional and adaptive [2]. When a person experiences loneliness in response to social isolation, that person might be more likely to be activated to go out and initiate or restore social relationships than people who do not experience loneliness in response to social isolation. In turn, people who do experience loneliness are more likely to survive and pass on their genes, because the likelihood of survival is greater in a social community in which food is shared and people are protected from outside threats through stable social relationships. From this point of view, experiencing levels of loneliness that are transient (i.e., state loneliness) is not necessarily negative, and may even have positive consequences as a person experiencing state loneliness may be motivated to actively seek social contact. In contrast, levels of loneliness that are chronic and enduring (i.e., chronic levels of trait loneliness) are found to have negative consequences, such as cardiovascular disease, sleep problems, and depression [3].

From an evolutionary point of view, it can be expected that state levels of loneliness have a genetic basis, as these feelings may be adaptive for survival. Yet genetic studies have exclusively examined trait levels of loneliness. Behavioral genetic studies have found that trait levels of loneliness are moderately heritable, with heritability estimates ranging from 45 to 55% in children [4], [5], 75% in adolescents [6], and 48% in adults [7]. Further molecular-genetic research has shown that several genes, amongst which the oxytocin receptor gene (*OXTR*), are related to trait levels of loneliness in adolescents [8], [9]. As no studies have examined the genetic basis of state levels of loneliness, the goal of the present study was to examine relations between a variant in the *OXTR* gene (rs53576) and state levels of loneliness in early adolescents.

### Oxytocin Receptor Gene

Oxytocin is a neuropeptide that is related to different types of social behavior [10]. The effects of oxytocin might be dependent on genetic variation in the oxytocin receptor gene (*OXTR*). A single nucleotide polymorphism (SNP) within this gene, rs53576, encodes for two allelic variants, the A allele and the G allele. Up to now, little is known about the functionality of this gene, and to our knowledge, no studies have examined whether the two variants of this gene are related to differences in oxytocin receptor functionality or receptor density. However, previous research has found relations with behavioral phenotypes. Results from studies showed the A allele to be related to less sensitive parenting [11], less empathy and higher levels of stress [12], less sociality [13], and less optimism and self-esteem [14], although the relation between *OXTR* and optimism was not replicated in a different sample [15]. In addition, A carriers displayed less nonverbal affiliative cues in social interaction and were rated as less prosocial than GG carriers [16]. In contrast, another study found that patients with unipolar depression who carried the GG genotype had higher levels of separation anxiety and fearful attachment, compared to patients carrying an A allele [17].

### OXTR Gene and Loneliness

Regarding trait levels of loneliness, two studies have examined relations with the *OXTR* rs53576 genetic variant. A study on adults found that AA carriers experienced slightly higher levels of loneliness than people carrying a G allele [8], which was only present for males, not females. Further, a study on adolescents found a sex difference throughout adolescence, in that girls carrying an A allele showed a steeper decline in loneliness, compared to girls carrying the GG genotype [9]. These findings indicate that the AA genotype conveys a risk for loneliness in male adults [8], whereas the GG genotype can be considered a risk for relatively stable levels of loneliness throughout adolescence for girls [9].

As main effects of genes in mental disorders are often small and difficult to detect, it is important to operationalize the phenotype accurately and precisely. A solution for this problem may lie in examining micro-level effects of the *OXTR* gene. Therefore, we will examine state levels of loneliness in daily life, by using the Experience Sampling Method [18]. In this way, adolescents report on their actual feelings of loneliness in real life. The main advantages of this method are that (a) it prevents recall bias because participants report on what they are experiencing at that moment and (b) the ecological validity is high, as participants fill out the questionnaires in their natural environment.

### Gene-Environment Interactions

In a recent review, Bartz et al. [19] argue that oxytocin affects the perception of and attention to social cues. This could indicate that individuals with higher oxytocin levels are more sensitive to social cues, and hence also more affected by those social cues than individuals with lower oxytocin levels. These findings concur with the Differential Susceptibility Theory, which states that some individuals are more susceptible to their environment due to neurobiological factors [20]. Hence, in the present study we will examine gene-environment interactions as well.

The only study examining GxE interactions with the rs53576 variant of the *OXTR* gene in relation to trait loneliness [9] did not find any interactions. Regarding other outcomes findings are mixed. Two studies found that GG genotypes were more affected by their environment, in that people carrying the GG genotype experienced more negative outcomes (i.e., lower maternal sensitivity [21]; higher emotion dysregulation and disorganized attachment [22]) when they experienced more negative environments (i.e., high levels of interparental conflict or maltreatment), and experienced more positive outcomes when they experienced more positive environments (low levels of interparental conflict or maltreatment). These studies indicate that GG genotypes may be more negatively affected by negative environments than A carriers. In addition, another study found that G carriers (i.e., GG and AG genotypes) showed lower stress responses after receiving social support, compared to AA genotypes who did not benefit from social support [23]. In contrast, another study examining physical health problems found that for A carriers, exposure to more stressful life events was related to new-onset ailments, whereas there was no relation between stressful life events and ailments in GG genotypes [24]. These findings indicate that A carriers would be more susceptible to negative environments than GG genotypes.

In the present study, we will examine state-level gene-environment interactions. Previous research on state levels of positive and negative affect has shown that the perceptions adolescents have of their company (i.e., positive or negative) are related to their levels of positive and negative affect [25]. Therefore, we expect that adolescents’ perceptions of their company will be related to state levels of loneliness. Hence, we will examine whether positive and negative perceptions of company are related to state levels of loneliness, and whether these relations are moderated by the *OXTR* genotype.

### Sex Differences

Oxytocin receptors are partly upregulated by estrogen (e.g., [26]), a sex hormone which is particularly present in females. This can explain why some studies reported sex differences in the effects of the *OXTR* gene. For example, Tost and colleagues [13] found that the relation between *OXTR* and the brain volume of the hypothalamus and amygdala differed between males and females. Lucht et al. [8] showed that the relation between *OXTR* and positive affect was only present for males, but not for females. In Kogan et al. [16], marginally significant differences in prosociality were found between male and female GG genotypes. Importantly, some other studies did not find any sex differences [12], [14], or examined females only [11], [21]. Regarding trait levels of loneliness, it was found that the relation between the *OXTR* gene and the development of loneliness in adolescence was only present for girls [9]. Because of these sex-dependent findings, it is important to consider sex differences in the effects of the *OXTR* gene.

### The Present Study

The main aim of this study was to examine relations between the *OXTR* rs53576 genotype and state levels of loneliness. As previous findings on the associations between this *OXTR* genotype and trait levels of loneliness were inconsistent, we did not have specific hypotheses regarding direct relations between *OXTR* and state levels of loneliness. Second, we examined gene-environment interactions between perceptions of company and *OXTR* on state levels of loneliness. As previous studies have shown that GG genotypes were more negatively affected by negative environmental factors [21], [22], we hypothesized that the relation between negative perceptions of company would be stronger for adolescents carrying the GG genotype. As no studies have examined GxE interactions with positive environmental factors (e.g., the GxE studies described earlier only examined the absence of negative environments such as conflict or maltreatment), we did not have a specific hypothesis for the interaction between *OXTR* and positive perceptions of company.

Importantly, because adolescents are obliged to go to school during weekdays, the range of people whom they can choose to spend their time with is limited during weekdays. In weekends, on the other hand, adolescents can choose their company. This difference between week and weekend days may affect how adolescents perceive their company and the relations between these perceptions of company and state loneliness. Therefore, we also examined whether perceptions of company and state levels of loneliness differed between week and weekend days, and whether the interaction between *OXTR* and perceptions of company had different effects on state loneliness during week or weekend days. Finally, as mentioned before, because sex differences are present in the effects of the *OXTR* gene, all analyses were tested for boys and girls separately.

## Method

### Ethics Statement

The present study was approved by the Committee on Research Involving Human Subjects (CMO Arnhem-Nijmegen, 2009, No. 285). Both adolescents and their parents had to sign a consent form in order to participate in the study.

### Participants

The total sample consisted of 301 adolescents (39% boys) from four secondary schools. The age of the participants ranged between 13 and 16 years (*M = *14.19, *SD = *.55). The majority of the adolescents (97.1%) were born in The Netherlands and only 1.3% of the adolescents was not born in an European country. Educational levels were equally distributed (i.e., 24% preparatory secondary school for technical and vocational training, 36% preparatory school for college, 40% preparatory school for university).

### Procedure

Adolescents were recruited through high schools. Schools were sent information letters in which they were asked to participate in the present study. In schools that provided their consent, all second-year students and their parents received a letter in which they were asked to participate.

The study consisted of (a) a baseline questionnaire, (b) saliva collection for genetic analyses, and (c) the Experience Sampling Method (ESM) period. For a detailed description of the procedure, see [25]. The baseline questionnaire was administered online during school hours, after which they were asked to provide saliva (Oragene; DNA Genotek Inc., Ottawa, ON, Canada). The ESM period took place three to eight weeks after the baseline questionnaire and always started on Fridays. Adolescents received a smartphone, on which a program was installed (http://myexperience.sourceforge.net/) that emitted buzzing signals at nine random time points each day, for six consecutive days. When adolescents received a signal, they had to immediately pause their activity and fill out the questionnaire on the smartphone. Data were stored on the smartphones and a text message was sent to the principal investigator after each completed questionnaire, making it possible to check compliance. Adolescents received the full reward of € 20 (i.e., about 27 US $) when they completed at least 55% of the momentary assessments.

### Materials

#### State loneliness

We used four items to measure state levels of loneliness: lonely, isolated, left out, and abandoned. Adolescents had to rate at each momentary assessment to what extent they experienced the described emotion on a 7-point scale, ranging from (1) *not at all* to (7) *very much*. Cronbach’s alpha was calculated for each momentary assessment separately, and then averaged over all momentary assessments, which resulted in an alpha of.73. Inter-item correlations ranged from *r* = .43 to *r* = .90.

#### Perceptions of company

When adolescents reported that they were with other people at the time of the ESM signal, positive and negative perceptions of company were measured (from now on referred to as ‘positive company’ and ‘negative company’). Positive company consisted of the items “I feel accepted by this company” and “I feel comfortable in this company” (*r* = .60). Negative company consisted of the items “I feel threatened by this company” and “I feel judged by this company” (*r = *.37).

#### OXTR genotyping

DNA was isolated from saliva using the Oragene system (DNA Genotek Inc., Kanata, Ontario, Canada). The *OXTR* polymorphism rs53576 was genotyped using Taqman analysis (Taqman Allelic Discrimination assay ID: C___3290335_10, reporter 1: VIC-A-allele, forward assay; Applied Biosystems, Nieuwerkerk a/d IJssel, The Netherlands). Genotyping was carried out in a volume of 5 µl containing 10 ng of genomic DNA, 2.5 µl of Taqman Mastermix (2x; Applied Biosystems) and 0.0625 µl of the Taqman assay (40x) and 1.4375 µl of MilliQ. Each amplification was performed by an initial denaturation at 95°C for 12 min, followed by 40 cycles of denaturation at 92°C for 15 seconds and annealing/extension at 60°C for 1 min. Genotyping was performed on a 7500 Fast Real-Time PCR System. Genotypes were scored using the algorithm and software supplied by the manufacturer (Applied Biosystems). Genotyping was performed in a laboratory at the Department of Human Genetics of the Radboud University Nijmegen Medical Centre in Nijmegen, which is accredited by the leading institute for laboratories in the health care sector in the Netherlands (called CCKL). Generally, 5% blanks as well as duplicates between plates were taken along as quality controls during genotyping. No deviations from Hardy-Weinberg equilibrium (HWE) were detected (*p = *.87). To maximize the power of the analyses, the *OXTR* genotype was dummy coded into 0 (GG) and 1 (AA/AG).

### Power Analysis

We conducted power-analyses using the software Quanto to test whether we had enough power for the gene-environment interactions [27]. For the power calculation we applied the gene-environment design option for continuous outcomes with independent individuals. Further, it was assumed that approximately 30% of the sample would have the *OXTR*-A allele [8]. The assumed inheritance model was dominant. Finally, the assumed main effects of the *OXTR* genotype and negative company were 0.02 and 0.18, respectively. To detect a small effect for the gene by sex interaction with an *R* ^2^ of 0.02 to 0.03, with 80% power (alpha = .05), the sample size required is between 205 and 310. For positive company (assumed main effect was 0.13), we needed a sample size between 218 and 330 to detect a small effect of the gene-environment interaction with an *R* ^2^ of 0.02 to 0.03 and 80% power (alpha = .05). This indicates that with our sample size of 275 adolescents, we had enough power to detect a small effect size of the *OXTR* x company interactions.

### Statistical Analyses

We examined relations between the *OXTR* genotype and state levels of loneliness. Because our repeated momentary assessments (Level 1) were nested within individuals (Level 2), we conducted multilevel linear regression analyses in Mplus [28]. To examine possible sex differences in the relations between the *OXTR* genotype and state levels of loneliness, we conducted multi-group analyses across sex. We did this by examining whether the model fit for the model in which the paths were allowed to differ between boys and girls was significantly better than the model fit for the model in which the paths were constrained to be equal for boys and girls, using a chi-square difference test (Δχ^2^) [29]. If significant differences between boys and girls would emerge, we further compared differences between boys and girls per path, by examining whether the model fit of the model in which the path of interest was allowed to differ between boys and girls was better than the model fit for the model in which all paths were constrained, also by using the chi-square difference test.

Level 1 predictors (i.e., positive and negative company) were centered at group-level and were included in the model as random coefficients. In this way, it is possible to examine whether the relations between the Level 1 variables vary across adolescents [30]. Hence, by using this approach, we can examine whether the relation between the Level 1 variables (positive and negative) company and state loneliness varies across adolescents, by including positive and negative company as random predictors in the model. When these coefficients are significant, this implies that the relations between negative company and affect differ between adolescents, and can therefore be predicted by individual characteristics, such as the *OXTR* genotype.

First, we tested the empty model without predictors. Second, the *OXTR* genotype was added to the model to examine relations between the genotype and state loneliness. Third, we examined relations between positive and negative company and state loneliness, in two separate models. Fourth, the interactions between the *OXTR* genotype and negative and positive company were examined over all sampling days. Next, we split the analyses for week days and weekend days to further examine whether the interaction between *OXTR* and negative and positive company had different effects on state loneliness during week or weekend days.

Finally, as the inter-item correlation between the two negative company items was relatively low, we checked in additional analyses whether the results involving negative company differed between these two items (i.e., I feel judged by this company, I feel threatened by this company).

## Results

### Descriptive Statistics

**Figure 1:**
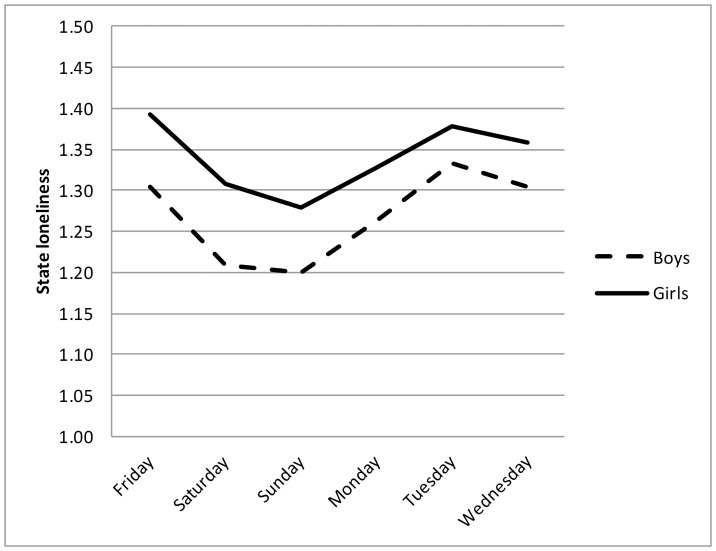
Levels of state loneliness during the ESM period, split for boys and girls.

Means and standard deviations for the model variables are depicted in Table 1, separately for boys and girls. Mean levels of state loneliness were relatively low, compared to the range (i.e., 1–7). In Figure 1, state levels of loneliness are depicted across the ESM period, split for boys and girls. As can be seen in this Figure, state levels of loneliness were lowest during weekend days (*t* [274] = 4.49, *p*<.001), for both boys and girls. For the *OXTR* genotype, 110 adolescents (41.9%) carried the GG genotype (43 boys and 67 girls), 122 adolescents (46.6%) carried the heterogenous genotype (51 boys and 71 girls), and 30 adolescents (11.5%) were homozygous for the A allele (12 boys and 18 girls). No sex differences were found for any of the variables.

**Table 1:** Descriptive Statistics, Split for Boys and Girls.

	Boys		Girls			
Variable	*M (SD)*	Range	*M (SD)*	Range	*t*	*df*
Stateloneliness^a^	1.30 (0.40)	1.00–2.97	1.36 (0.43)	1.00–3.31	−1.21	274
Positivecompany^a^	6.14 (0.65)	4.00–7.00	6.15 (0.57)	4.46–7.00	−0.10	274
Negativecompany^a^	1.52 (0.58)	1.00–3.41	1.56 (0.51)	1.00–4.58	−0.62	274

*Note*. ^a^For the momentary assessment data, aggregated mean scores were calculated within persons.

Next, correlations between model variables were examined (see Table 2). For boys, no significant correlations were found between the *OXTR* genotype and the model variables. In contrast, for girls we found small positive correlations between the *OXTR* genotype and state loneliness and negative company, indicating that A carriers experienced slightly higher levels of state loneliness and negative company than girls with the GG genotype. State levels of loneliness were negatively correlated with positive company and positively correlated with negative company in both boys and girls. As we found significant correlations between the *OXTR* genotype and state loneliness for girls, all subsequent analyses are tested in multigroup models, for boys and girls separately.

**Table 2:** Correlations Between Model Variables, Split for Boys and Girls.

Variable	1.	2.	3.	4.
1. *OXTR* genotype^a^	–	.20*	−.06	.19*
2. State loneliness	−.14	–	−.51**	.60**
3. Positive company	.07	−.67**	–	−.65**
4. Negative company	−.10	.70**	−.72**	–

*Note*. *p<.05;

** *p*<.001.

Above the diagonal correlations for girls, below the diagonal correlations for boys.

a 0 = GG, 1 = A/AG.

### Model Outcomes

In the next models, we tested the relation between the *OXTR* gene and state levels of loneliness. First, we tested the unconditional model without predictors. The intra-class correlation was.31 for girls and.35 for boys, indicating that 31–35% of the variation in state loneliness occurred at the individual level (Level 2). The variances in state loneliness were significant at the momentary assessment level (Level 1 variance boys = .24; Level 1 variance girls = .28) and at the individual level (Level 2 variance boys = .13; Level 2 variance girls = .13). We tested whether the constrained model differed from the unconstrained model, which was not the case (Δχ^2^ (2) = 1.44, *p*>.05), indicating that the unconditional model did not differ between boys and girls.

Subsequently, we entered the *OXTR* genotype as a predictor in the model (Level 2 model in Table 3). For boys, the relation between *OXTR* and state loneliness was not significant. In contrast, the relation between *OXTR* and state loneliness was significant for girls, in that A-allele carriers had higher levels of state loneliness than carriers of the GG genotype. The model in which the relation between *OXTR* and state loneliness was allowed to differ for boys and girls showed a significant improvement in model fit compared to the model in which this path was constrained to be equal across sex (Δχ^2^ (1) = 5.77, *p*<.05). Next, we examined the relations between negative company and state loneliness (Level 1 model in Table 3) and positive company and state loneliness (Level 1 model in Table 4). For both boys and girls, negative company was positively related to state loneliness, in that higher levels of negative company were related to higher levels of state loneliness. In addition, positive company was negatively related to state loneliness in both boys and girls, indicating that higher levels of positive company were related to lower levels of state loneliness. For both models, no differences were found between boys and girls (Δχ^2^ (1) = 1.58, *p*>.05 for negative company; Δχ^2^ (1) = 0.48, *p*>.05 for positive company).

**Table 3:** Multi-Group Multilevel Models for Relations Between OXTR Genotype, Negative Company, and State Loneliness.

	Boys					Girls				
			All days	Week days	Weekend days			All days	Week days	Weekend days
Parameter	Level-2	Level-1: random	Interaction	Interaction	Interaction	Level-2	Level-1: random	Interaction	Interaction	Interaction
*Regression coefficients*										
Intercept	1.31 (.07)***	1.23 (.03)***	1.27 (.07)***	1.29 (.07)***	1.19 (.05)***	1.25 (.03)***	1.29 (.03)***	1.22 (.03)***	1.26 (.04)***	1.14 (.03)***
Negative company		.10 (.02)***	.11 (.04)**	.14 (.04)**	.02 (.04)		.15 (.02)***	.15 (.03)***	.17 (.03)***	−.04 (.04)
*OXTR* genotype	−.08 (.08)		−.06 (.08)	−.07 (.08)	−.01 (.08)	.14 (.06)*		.12 (.05)*	.11 (.06)	.15 (.05)**
*OXTR* x Company			.00 (.05)	−.02 (.06)	.18 (.10)			.00 (.04)	−.01 (.05)	.17 (.06)**
*Variance components*										
Level-1 variance	.24 (.04)***	.14 (.03)***	.15 (.03)***	.17 (.03)***	.12 (.02)***	.28 (.03)***	.19 (.02)***	.19 (.02)***	.21 (.02)***	.12 (.02)***
Level-2 intercept variance	.13 (.03)***	.12 (.03)***	.12 (.03)***	.14 (.04)***	.09 (.02)***	.12 (.02)***	.11 (.02)***	.10 (.02)***	.11 (.02)***	.09 (.02)***
Level-2 slope variance		.04 (.01)**	.04 (.01)**	.04 (.01)**	.04 (.01)**		.03 (.01)***	.03 (.01)***	.05 (.01)***	.04 (.01)**
*Model difference test*										
Chi square	5.77*	1.58	6.06	6.37	5.62	5.77*	1.58	6.06	6.37	5.62
Df	1	1	4	4	4	1	1	4	4	4

*Note*. All observation-level variables were group-mean centered.

* *p*<.05.

** *p*<.01.

*** *p*<.001.

**Table 4:** Multi-Group Multilevel Models for Relations Between OXTR Genotype, Positive Company, and State Loneliness.

	Boys					Girls				
			All days	Week days	Weekend days			All days	Week days	Weekend days
Parameter	Level-2	Level-1: random	Interaction	Interaction	Interaction	Level-2	Level-1: random	Interaction	Interaction	Interaction
*Regression coefficients*										
Intercept	1.31 (.07)***	1.23 (.03)***	1.27 (.07)***	1.29 (.07)***	1.19 (.05)***	1.25 (.03)***	1.29 (.03)***	1.22 (.03)***	1.26 (.04)***	1.14 (.03)***
Positive company		−.08 (.02)***	−.10 (.04)**	−.09 (.04)*	−.04 (.05)		−.02 (.02)**	−.08 (.02)***	−.09 (.02)***	−.02 (.02)
*OXTR* genotype	−.08 (.08)		−.06 (.08)	−.07 (.08)	−.00 (.08)	.14 (.06)*		.12 (.05)*	.11 (.06)	.15 (.05)**
*OXTR* x Company			.04 (.04)	.03 (.05)	.05 (.10)			−.03 (.03)	−.03 (.03)	−.07 (.04)†
*Variance components*										
Level-1 variance	.24 (.04)***	.14 (.02)***	.14 (.02)***	.16 (.03)***	.06 (.02)***	.28 (.03)***	.20 (.02)***	.20 (.02)***	.23 (.03)***	.12 (.02)***
Level-2 intercept variance	.13 (.03)***	.11 (.02)***	.12 (.03)***	.14 (.04)***	.15 (.10)	.12 (.02)***	.11 (.02)***	.10 (.02)***	.11 (.02)***	.09 (.02)***
Level-2 slope variance		.01 (.00)***	.03 (.01)**	.04 (.01)**	.04 (.04)		.02 (.00)***	.02 (.00)***	.02 (.01)**	.03 (.02)
*Model difference test*										
Chi square	5.77*	0.48	8.34	7.09	7.39	5.77*	0.48	8.34	7.09	7.39
Df	1	1	4	4	4	1	1	4	4	4

*Note*. All observation-level variables were group-mean centered.

† <.07.

* *p*<.05.

** *p.*

**Figure 2:**
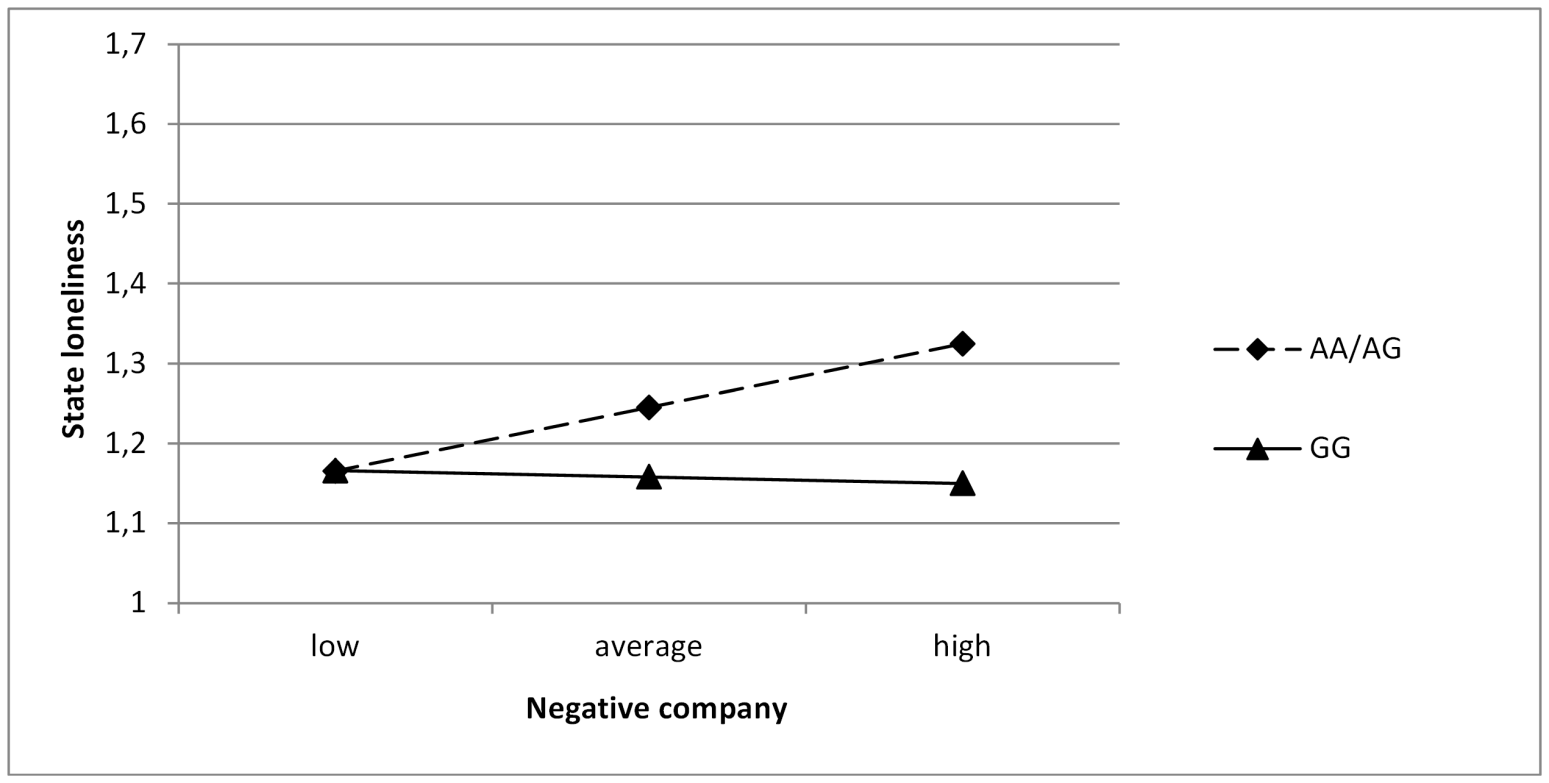
Relation between negative company and state loneliness on weekend days for girls, split for the OXTR genotype.

In the next models, the interactions between the *OXTR* genotype and positive and negative company were examined (see Table 3 for negative company and Table 4 for positive company). No significant gene-environment interactions were found, and no sex differences were found (Δχ^2^ (4) = 6.06, *p*>.05 for negative company; Δχ^2^ (4) = 8.34, *p*>.05 for positive company). However, when we split the analyses for weekdays and weekend days, we did find a significant gene-environment interaction between *OXTR* and negative company for girls on weekend days only. When comparing the constrained model with the unconstrained model, it was found that this model did not significantly differ between boys and girls (Δχ^2^ (4) = 5.62, *p*>.05). Further analyses showed that this interaction was present in the total sample (*B* = .15, *SE* = .05, *p*<.01). This finding indicated that adolescents carrying an A-allele were more negatively affected by negative company on weekend days, in that they experienced higher levels of state loneliness when they perceived their company more negatively, than adolescents carrying the GG genotype (see Figure 2). For positive company, no significant gene-environment interactions were found, and no differences were found between boys and girls (see Table 4).

### Additional Analyses

In the present study, a dominant model for the A allele was assumed. Because of the small group of adolescents with the AA genotype (*N* = 30), it was not possible to examine differences in results between the three genotypes. However, we could examine whether the results would differ if we included only adolescents with GG genotypes versus adolescents with the AG genotype. No differences were found in these analyses, indicating that our results were not due to the small group of adolescents with the AA genotype.

Due to the low inter-item correlation between the two negative company items (“I feel judged by this company” and “I feel threatened by this company”), we conducted additional analyses to examine whether we would find different results for the gene-environment interactions when we included these items separately in the models. For the models in which we examined interactions between negative company and *OXTR* on state loneliness on weekdays and on the total sampling period, no differences were found between the items. That is, all gene-environment interactions were non-significant, as was found for the combined negative company measure. However, when we examined the gene-environment interaction on weekend days only, we found different results for the two items. For the item measuring threat, no significant interaction was found, neither for boys (*B = *0.16, *SE = *0.20, *p*>.05), nor for girls (*B* = 0.09, *SE = *0.09, *p*>.05). When we examined the interaction for the item reflecting feelings of being judged, we found a marginally significant effect for boys (*B = *0.11, *SE = *0.06, *p = *.06), and a significant effect for girls (*B = *0.13, *SE = *0.04, *p = *.001). Model fit comparisons showed that the constrained model did not differ from the unconstrained model (Δχ^2^ (4) = 3.07, *p*>.05), which indicated that the interaction does not differ between boys and girls. Hence, adolescents carrying an A allele experienced higher levels of state loneliness when they felt more judged by their company, compared to adolescents carrying the GG genotype (*B = *0.14, *SE = *0.03, *p<*.001).

## Discussion

The present study aimed to investigate relations between the *OXTR* genotype, perceptions of company, and state levels of loneliness. We found a significant relation with the *OXTR* rs53576 variant for girls, in that girls carrying an A allele had significantly higher levels of state loneliness than girls carrying the GG genotype. No significant interactions were found for perceptions of company and *OXTR* on state loneliness. However, when the analyses were split for week and weekend days, we did find significant gene-environment interactions. These results indicate that adolescents carrying an A allele may be more susceptible for negative environments, during weekend days.

### Main Effects OXTR Gene

We found a significant relation between state loneliness and the *OXTR* rs53576 variant in girls, in that girls carrying at least one A allele experienced higher levels of state loneliness than girls carrying the GG genotype. These findings are in line with previous studies showing that the A allele is related to maladaptive outcomes [11], [12], [13], [14], [16]. Our findings are in contrast with results from van Roekel et al. [9], who found the GG genotype to be related to stable levels of trait loneliness in adolescence. Importantly, as trait and state levels of loneliness are distinct phenotypes, it is difficult to compare these results.

### Gene-Environment Interactions

We found no significant gene-environment interactions between positive and negative company on state loneliness when we analyzed all days together. Yet, when we examined the relations for week and weekend days separately, we found significant interactions on weekend days only. These findings showed that adolescents carrying an A allele had higher levels of state loneliness when they perceived their company more negatively, whereas state loneliness in GG genotypes was not affected by negative company. This interaction is in line with a diathesis-stress model [31], [32], which states that dual risks, that is, carrying a ‘risk’ allele (i.e., the A allele) and experiencing negative environments (i.e., negative company) lead to most negative outcomes. Interestingly, we only found this gene-environment interaction for weekend days. A possible explanation may be that the company adolescents were in had a greater impact on them during weekends. In weekends, adolescents most often are free to choose who they want to spent their time with and how they want to spent their time, whereas on weekdays, they are less free to choose their company. Therefore, their perceptions of the company they are with during weekends may have a greater impact on their feelings of loneliness. The importance of weekends is also accentuated by previous research, in which it was found that adolescents experience the highest levels of state loneliness when they are alone at weekend days, and more specifically, Friday and Saturday nights [33]. The perceptions of company adolescents have during weekends may affect their loneliness levels more than the perceptions of company adolescents have during week days.

To our knowledge, only three other studies found gene-environment interactions with the *OXTR* rs53576 variant on maternal sensitivity [21], emotion dysregulation [22], and physical health problems [24]. Two studies found that GG carriers were more susceptible for negative environments (i.e., interparental conflict and childhood maltreatment) [21], [22], whereas the study on physical health problems found that A carriers were more susceptible to negative environments [24]. The results from Bradley et al. and Sturge-Apple et al. are in contrast with our findings, which showed that adolescents carrying an A allele were more negatively affected by their environment. Importantly, it is difficult to compare our findings to those of Bradley et al. and Sturge-Apple et al., because the outcome variables, the definitions of the environment, and the designs of the studies are not comparable. In our study, participants rated their environment when they were actually in it, whereas in the previous studies, participants had to report on their environment retrospectively. Therefore, it could be that GG carriers are more negatively affected by very negative, retrospectively rated environments, whereas A carriers are more affected by negative environments, at the moment that they experience those environments. These contradictory findings indicate that further research on gene-environment interactions with the *OXTR* gene is warranted.

Our findings indicate that adolescents carrying an A allele may be more susceptible to their environment. These effects were only found on a micro-level, which may explain why we found opposite results to the study of van Roekel et al. [9]. As adolescents carrying the GG genotype are less susceptible to their direct, real-life environment, which was found in the present study, their levels of trait loneliness may remain more stable, because these levels are not greatly affected by their environment, as was found in the study by van Roekel et al. [9]. In contrast, adolescents carrying an A allele may be more affected by their environment and therefore decrease in trait loneliness over time. As we did not have longitudinal data, we could not examine this assumption in our sample. Further research is necessary to explain the differences between the previous findings on trait loneliness and the present findings on state loneliness. A possible solution for this contradiction in findings may be to examine genetic effects and gene-environment interactions on both trait and state levels of loneliness in a longitudinal design, in which adolescents annually fill out trait loneliness questionnaires, and several ESM periods take place between successive annual waves. In that way, it is possible to examine genetic effects and gene-environment interactions on state and trait loneliness simultaneously.

In our additional analyses in which we checked whether the results for negative company differed between the two items (i.e., ‘I feel judged by this company’ and ‘I feel threatened by this company’), we showed that the significant gene-environment interaction with negative company on weekend days was only present for the ‘judged’ item, and not for the ‘threatened’ item. This indicates that A carriers respond more negatively to perceptions of judging company, and not to perceptions of threat. A possible explanation for this may be that in general, adolescents more often experience being judged by their company than being threatened by their company and therefore, differences between genotypes may become visible. However, further research is necessary to disentangle these different effects.

### Sex Differences

Several explanations can be given for the sex differences in the findings of the present study. As was mentioned before, oxytocin receptors are affected by levels of estrogen, a sex hormone that is particularly present in females. Therefore, variation in this gene might be more relevant in girls. Importantly, sex differences are common in research on the *OXTR* rs53576 variant [11], [13], but are not always found [12], [14]. Also, in this study the difference patterns were not consistent, warranting more research on sex differences in the effects of the *OXTR* gene.

### Strengths and Limitations

One of the major strengths of the present study is that we examined gene-environment interactions in ‘the real world’. Although it is important that these results are replicated, our findings provide greater insight into the role of the *OXTR* gene in internalizing problems. In addition, because our measures are administered in daily life, our findings could provide more appropriate starting points for intervention and prevention than trait-level variables do.

A first limitation of the present study is that our sample size was relatively small, which might imply that we did not have sufficient power to find significant relations. However, our power increased as we measured our outcome variable as well as the environment multiple times, resulting in more reliable measures, which in turn increased power. This is also substantiated by the results from the power analyses, showing that we had enough power to detect a gene-environment interaction.

Second, because we only examined relations cross-sectionally, it is not possible to determine the direction of effects. It could be that the perceptions of company predict subsequent levels of state loneliness, but it is also possible that experiencing loneliness influences adolescents’ perceptions of company. Importantly, irrespective of the direction of effects, our results showed that the *OXTR* gene moderated these relations.

Third, mean levels of state loneliness were relatively low in our sample. Because the few studies that have examined state levels of loneliness did not report on mean levels, we could not compare our mean levels on state loneliness with other studies. However, mean levels of negative affect in other studies on early adolescents (i.e., anxiety, depressive feelings, and irritation [34]) are comparable to our mean levels on state loneliness, which may indicate that these levels are not extraordinarily low.

Fourth, previous research showed that adolescents experienced more positive affect when they were with friends, compared to when they were with family [35], which indicates that the type of company adolescents are in affects their feelings. It is possible that in our sample, adolescents were more susceptible to a specific type of company. Splitting the analyses for these subgroups was not possible in the present study, as this would have resulted in very small groups. Future research could examine whether the type of company can explain why adolescents with an A allele were more affected by negative perceptions of company.

Fifth, currently we do not know what the functionality of the *OXTR* rs53576 variant is in terms of gene expression and actual oxytocin levels. Hence it could be that our findings are caused by another SNP that is in linkage disequilibrium with the rs53576 variant. Further research is necessary to examine the function of the *OXTR* rs53576 variant.

## Conclusions

To conclude, the present study showed that girls carrying an A allele had higher levels of state loneliness than girls carrying the GG genotype. In addition, both boys and girls with an A allele were more negatively affected by negative company than boys and girls with the GG genotype, on weekend days only. Our findings highlight the importance of operationalizing the outcome variable and the environmental variables precisely and accurately, as we only find gene-environment interactions on weekend days.
